# Neurologic Manifestations of Coronavirus Disease 2019 in Children: An Iranian Hospital-Based Study

**DOI:** 10.34172/aim.2023.25

**Published:** 2023-03-01

**Authors:** Elmira Haji Esmaeil Memar, Morteza Heidari, Homa Ghabeli, Elham Pourbakhtyaran, Roya Haghighi, Seyyed Mohammad Mahdi Hosseiny, Setareh Mamishi, Shima Mahmoudi, Hamid Eshaghi, Ali Reza Tavasoli, Mahmoud Mohammadi, Reza Shervin Badv, Gholamreza Zamani, Masood Ghahvehchi Akbari, Bahram Yarali, Rohola Shirzadi, Masoud Mohammadpour, Bahareh Yaghmaei, Meisam Sharifzadeh Ekbatani, Zeynab Najafi, Mahmoud Reza Ashrafi

**Affiliations:** ^1^Pediatrics Center of Excellence, Children’s Medical Center, Tehran University of Medical Sciences, Tehran, Iran; ^2^Department of Pediatric Neurology, Pediatrics Center of Excellence, Children’s Medical Center, Tehran University of Medical Sciences, Tehran, Iran; ^3^Myelin Disorders Clinic, Pediatric Neurology Division, Children’s Medical Center, Pediatrics Center of Excellence, Tehran University of Medical Sciences, Tehran, Iran; ^4^Department of Infectious Diseases, Pediatrics Center of Excellence, Children’s Medical Center, Tehran University of Medical Sciences, Tehran, Iran; ^5^Pediatric Infectious Disease Research Center, Tehran University of Medical Sciences, Tehran, Iran; ^6^Department of Physical Medicine and Rehabilitation, Pediatrics Center of Excellence, Children’s Medical Center, Tehran University of Medical Sciences, Tehran, Iran; ^7^Pediatric Respiratory and Sleep Medicine Research Center, Children’s Medical Center, Tehran University of Medical Sciences, Tehran, Iran; ^8^Department of Pediatric Intensive Care, Pediatrics Center of Excellence, Children’s Medical Center, Tehran University of Medical Sciences, Tehran, Iran; ^9^Pediatric Cell and Gene Therapy Research center, Gene, Cell &Tissue Research Institute, Tehran University of Medical Sciences, Tehran, Iran

**Keywords:** Children, COVID-19, Neurological manifestations, SARS-CoV-2

## Abstract

**Background:**

COVID-19 infection and its neurological manifestations were seen in children although less common than adults. The aim of this study was to determine the frequency of different types of neurologic findings of hospitalized children with COVID-19.

**Methods:**

This retrospective study was performed on hospitalized pediatric patients aged≤18 years with confirmed SARS-CoV-2 at Children’s Medical Center Hospital. Neurological manifestations were defined as the presence of any of the following symptoms: seizure, altered mental status, behavioral/personality change, ataxia, stroke, muscle weakness, smell and taste dysfunctions, and focal neurological disorders.

**Results:**

Fifty-four children with COVID-19 were admitted and their mean age was 6.94±4.06 years. Thirty-four of them (63%) were male. The most frequent neurological manifestation was seizure (19 [45%]) followed by muscle weakness (11 [26%]), loss of consciousness (10 [23%]), and focal neurological disorders (10 [23%]). Other neurological manifestations consisted of headache (n=7), movement disorders (n=6), behavioral/personality change (n=5), ataxia (n=3), and stroke (n=3). Twenty-nine percent of our patients had leukocytosis. A neutrophil count above 70% was seen in 31% of participants. Among our patients, 81% had a positive reverse-transcription polymerase chain reaction (RT-PCR) test for SARS-CoV-2.

**Conclusion:**

During the current pandemic outbreak, hospitalized children with COVID-19 should be evaluated for neurological signs because it is common among them and should not be under-estimated.

## Introduction

 SARS-CoV-2 was first found in December 2019 in Wuhan, China. This syndrome is mediated by a novel virus from the *Coronaviridae* family.^1^ SARS-CoV-2 is a beta virus, an envelope virus with a helical nucleocapsid and indeterminate positive ribonucleic acid that can infect mammals.^2,3^

 At the end of 2020, there were more than 80 million confirmed cases and roughly 2 million fatalities from COVID-19.^4^

 Many non-respiratory manifestations were identified in COVID-19 patients. Although there have been fewer studies with a large sample size reporting neurological abnormalities in COVID-19 patients, findings of neurological features are increasing. Common neurological symptoms of the disease include muscle pain, headaches, and loss of taste (dysgeusia), as well as anosmia, a loss of smell.^5,6^ New research has demonstrated that SARS-CoV-2 can manifest as potentially fatal neurological abnormalities such as ischemic stroke, acute demyelinating encephalomyelitis, and subarachnoid hemorrhage.^7-9^

 Neurological manifestations have also been reported in Middle East respiratory syndrome coronavirus (MERS-CoV) and SARS-CoV.^10,11^

 Children with COVID-19 may present neurologic symptoms, notwithstanding rarity (COVID-19). The purpose of this study was to evaluate the prevalence of various neurological manifestations in COVID-19-positive hospitalized children.

## Materials and Methods

###  Study Design and Population 

 This retrospective study was conducted on hospitalized pediatric patients under the age of 18 with confirmed SARS-CoV-2 infection or multi-system inflammatory syndrome in children (MIS-C) at Children’s Medical Center Hospital in Tehran, Iran, between February 1, 2020, and January 31, 2021.

 SARS-CoV-2 infection was confirmed by detection of the virus in a clinical specimen (nasopharyngeal swab, throat swab, nose swab, endotracheal tube aspirates) by reverse-transcription polymerase chain reaction (RT-PCR).^12^ The criteria of the Centers for Disease Control and Prevention (CDC) were used to determine MIS-C^13^ with an additional modification to include those with fever for less than 2 days if corticosteroids or intravenous immunoglobulin (IVIG) was administered before day 3 of fever.

 All clinical and laboratory investigations were performed according to institutional protocols.

###  Clinical Data Collection and Case Definitions 

 Chart review was performed by research study personnel at this participating center using a standardized case report form. Study data were collected which included demographics, medical history, clinical data, laboratory investigations, neuroimaging, and treatment modalities.

 Neurological manifestations were defined as the presence of any of the following symptoms: seizure, altered mental status, behavioral/personality change, ataxia, stroke, muscle weakness, smell and taste dysfunctions, focal neurological disorders. Regarding the previous condition of patients in neurodevelopment and other children had a history of epilepsy before COVID-19 infection and the rest were normal without any underlying diseases except FC patient.

###  Statistical Analysis

 The data were analyzed using SPSS version 24, (SPSS Inc., Chicago, IL). Statistical significance was defined as a *P* value < 0.05. For qualitative and quantitative variables, descriptive data were reported as percent (frequencies) or as mean (SD), respectively. Student’s *t* test was used to compare continuous and parametric data between groups, while the Mann-Whitney U test was used to compare nonparametric data for variables without a normal or standardized distribution. The *t* test was used to examine categorical data.

## Results

###  Demographics 

 Out of 943 patients admitted from February 01, 2020 to January 31, 2021, a total of 239 patients had COVID-19 disease and 54 of them had neurological symptoms. The mean age of the patients was 6.94 ± 4.06 years, and 34 (63%) were male. Non-neurological clinical manifestations of the participants are shown in Table 1.

**Table 1:** Non-neurological Clinical Manifestations of Participants (n = 42)

**Clinical Features**	**Frequency**	**Percent**
Fever	25	59
Body contusion	0	0
Cough	1	2
Dyspnea	1	2
Sore throat	1	2
Myalgia	3	7
Fatigue	1	2
Conjunctivitis	0	0
Diarrhea	10	23
Vomiting	4	9
Chills	4	9
History of smell disorder	0	0
History of taste disorder	0	0
History of strabismus	0	0
History of headache	39	93
History of vision disorder	41	98
Lung CT scan		
No CT	39	93
> 50% involvements	2	5
< 50% involvements	1	2

###  Neurological Clinical Manifestations and Complications of Participants

 The most frequent neurological manifestation was seizure (19 [45%]) followed by muscle weakness (11 [26%]), loss of consciousness (LOC) (10 [23%]), and focal neurological disorder (10 [23%]).

 Other neurological manifestations consisted of headache (n = 7), movement disorders (n = 6), behavioral/personality change (n = 5), ataxia (n = 3), and stroke (n = 3) (Table 2).

**Table 2:** Neurological clinical manifestations and complications of participants (n = 42)

**Neurological manifestations and complications**	**Frequency**	**Percent**
Headache	7	17
Vertigo	2	5
LOC	10	24
Smell and taste dysfunctions	0	0
Seizure	19	45
Behavioral/personality change	5	12
Muscle weakness	11	26
Ataxia	3	7
Stroke	3	7
Focal neurological disorders	10	24
Movement disorders	6	14

LOC, loss of consciousness.

###  Laboratory Findings

 Twenty-ninepercent of our patients showed leukocytosis. A neutrophil count above 70% was seen in 31% of participants. Among our patients, 81% had a positive SARS-CoV-2 RT-PCR test (Table 3).

**Table 3:** Laboratory data of participants (n = 42)

**Laboratory Data**		**Frequency**	**Percent**
WBC ( × 10^6^ cells per L)	< 4000	1	2
	4000–10000	15	36
	> 10000	12	29
Neutrophil ( × 10^6^ cells per L)	< 50%	9	21
	50–70%	12	29
	> 70%	13	31
Lymphocyte (%)	< 20%	11	26
	20–40%	15	35
	> 40%	7	16
Hemoglobin (g/dL)	< 11	10	24
	11–16	26	62
	> 16	1	2
PLT ( × 10^6^ cells per L)	< 150	2	5
	150–450	34	81
	> 450	2	5
Ferritin (ng/mL)	< 30	1	2
	30–220	10	24
	> 220	4	10
CPK (IU/L)	< 24	1	2
	24–172	16	38
	> 172	6	14
CRP (mg/L)	> 6	14	33
	< 6	20	48
ESR (mm/h)	> 10	15	36
	< 10	20	48
LDH (U/L)	< 5	17	41
	5-850	24	57
BE	< -2	19	45
	-2 < n < + 2	1	2
HCO_3_ (mEq/L)	< 22	17	41
	22–26	4	9
PCO_2_ (mm Hg)	< 35	12	29
	35–45	9	21
PH	< 7.35	6	14
	7.35–7.45	14	33
	> 7.45	1	2
SARS-CoV-2 RT-PCR	Positive	34	81
	Negative	7	17
SARS-CoV-2 IgM	No test	27	64
	Negative ( < 5)	15	36
SARS-CoV-2 IgG	No test	26	62
	Negative ( < 5)	4	10
	Borderline (5–10)	5	12
	Positive ( > 10)	7	17

CRP, C-reactive protein; PLT, Platelet; CPK, Creatine Phosphokinase; ESR, Erythrocyte sedimentation rate; LDH, Lactate dehydrogenase; BE, Base excess.

## Discussion

 The third coronavirus outbreak, following MERS in 2012 and SARS in 2002, is called COVID-19. One of the most significant organs affected by these viruses is the nervous system.^14,15^

 One new report suggests that some children with epilepsy may be at greater risk for COVID-19 involvement, particularly those who also have other medical complications and drug-resistant epilepsy. Thus, it is recommended that all child neurologists and caregivers should use techniques to reduce the risk of COVID-19 in these predisposed children.^16^

 Myalgia (28.5%) and headaches (14%) were reported in one systematic literature review as usual signs of COVID-19.^17^ In contrast to this study, among hospitalized patients, headache was an independent predictor of lower risk of mortality.^18^

 According to another study, a common manifestation of COVID-19 was severe headaches with the migraine phenotype, especially its bilateral form.^19^ Unlike previous studies, 17% of our patients had headaches.

 The prevalence of other neurological manifestations in hospitalized pediatrics with COVID-19 was evaluated in our study; after seizure and muscle weakness, LOC was in the third place of the most common symptoms. The seizure was repeatedly neurological manifestation in half of our patients.

 Direct virus attack or subsequent para-infectious pathways may be to blame for the nervous system’s involvement in COVID-19. Angiotensin-converting enzyme receptor receptors,^20^ which can be present on the nasal cup and ciliated epithelial cells and also oligodendrocytes, were discovered to be the mechanism by which SARS-CoV-2 penetrates human cells.^21^ SARS-CoV-2 can be found in the cerebrospinal fluid (CSF) of patients with encephalopathy, suggesting that the virus may be neuroinvasive ^22^ or demyelinating.^23^ Also, viral particles of SARS-CoV-2 were observed in the brain tissue of infected patients.^24^ It is important that these results should be taken with caution.^25^ However, some analyses on COVID-19 patients without neurological symptoms found no signs of SARS-CoV-2 in the brain tissue or CSF.^26^

 “Cytokine storm” which refers to the hyper-inflammatory response brought on by excessive cytokine synthesis (e.g., tumor necrosis factor and interleukin-6), is another potential major cause of neural damage in COVID-19.^4^

 Although in our study, no patient had a smell or taste dysfunction, COVID-19 patients in numerous studies were reported to have hyposmia/anosmia with a prevalence of about 20 to 98%.^5,6,27,28^ In non-hospitalized individuals, anosmia was more common than hospitalized COVID-19 patients.^29,30^ According to a recent study which was done on 576 patients, anosmia can lower the rate of mortality and decrease the severity of the disease.^31^ In an observational cohort research, more than 70% of COVID-19 patients with hyposmia and anosmia experienced early recovery 4 weeks following the onset of symptom.^32^ Methodological differences may be the cause of the differences in findings. Results may be greatly influenced by the study’s design, sample size, recruiting method (outpatients vs. inpatients), and way of evaluating smell perception (physical examination vs. self-reporting assessment).

 Cerebrovascular events such as intracranial hemorrhage (ICH), cerebral venous thrombosis, and ischemic stroke were reported in 0.5 to 1.3% of COVID-19 patients.^8,24^ Other effective parameters on early mortality were coincidence of COVID-19 and stroke, older age, higher baseline National Institutes of Health Stroke Scale, and cryptogenic stroke.^33^

 A few cases of post-infectious demyelinating disorders of the central nervous system (CNS) following COVID-19 illness have been described.^7,34^ Further longitudinal research should evaluate the frequency of these episodes in cases with COVID-19 that have been identified. Following recovery from COVID-19 disease, reports of post-infectious demyelination of the peripheral nervous system were also observed.^35^

 In our study, one case developed encephalitis, two patients had transverse myelitis and one patient had aseptic meningitis. In addition, GBS was another complication that involved five patients in our study. Bell’s palsy was observed in two of our participants. It was observed in approximately half the patients of the previous report.^36^

 According to the authors of one recent study,^37,38^ well-planned multicenter studies are required from several Iranian and international locations that closely track the various neurologic manifestations of this infection, utilizing neuroimaging studies, CSF studies, and neurophysiologic studies. Additionally, some of these severe neurologic consequences may be curable, such as non-convulsive seizures that cause agitation and are impossible to identify without regular electroencephalography testing.^37,38^

 This is, as far as we know, one of the few studies about the neurological manifestations of COVID-19 in children. Our study was subject to some limitations. Instead of the community, participants were chosen from the hospital (hospital-based vs. population-based studies). Selection bias may occur as a result. After the COVID-19 pandemic, Iran was among the countries at the top list of infection; so multicenter studies from all provinces of Iran are very important for investigating neurological complications. Some other limitations include absence of control group and the short follow-up duration. Therefore, more studies should be done to evaluate the occurrence of central nervous system demyelinating involvements and coincident parosmia and phantosmia (changed smell sensations).

 In conclusion,investigating COVID-19 patients, especially those children with simultaneous neurological symptoms, showed that we should noticed about underlying problems during our treatment and follow up for example if we have a child who has diabetes, we should notice about his/her diabetes to decrease neurological sequels.
